# Protective Effect of Thai *Perilla frutescens* Seed Oil Against Chronic Obstructive Pulmonary Disease Induced by Cigarette Smoke Extract in a Mouse Model

**DOI:** 10.1002/fsn3.71994

**Published:** 2026-06-05

**Authors:** Gaewarin Liamvilairat, Sorachai Srisuma, Julalux Thongam, Aurawan Kringkasemsee Kettawan, Aikkarach Kettawan, Thanaporn Rungruang

**Affiliations:** ^1^ Department of Anatomy, Faculty of Medicine Siriraj Hospital Mahidol University Bangkok Thailand; ^2^ Department of Physiology, Faculty of Medicine Siriraj Hospital Mahidol University Bangkok Thailand; ^3^ Institute of Nutrition Mahidol University Nakhon Pathom Thailand

**Keywords:** alveolar enlargement, chronic obstructive pulmonary disease (COPD), cigarette smoke extract (CSE), lung disease, *Perilla frutescens* seed oil (PSO)

## Abstract

Chronic obstructive pulmonary disease (COPD) is a progressive inflammatory lung disorder primarily caused by exposure to cigarette smoke. This leads to oxidative stress and alveolar damage. Current treatment strategies are largely palliative, focusing on symptom relief rather than halting disease progression. *Perilla frutescens* seed oil (PSO), which is rich in alpha‐linolenic acid, possesses notable antioxidant and anti‐inflammatory properties. However, its therapeutic potential in COPD remains inadequately explored. This study aimed to investigate the protective effects of Thai PSO on lung inflammation, oxidative stress, and histopathological changes in a mouse model of COPD induced by cigarette smoke extract (CSE). C57BL/6J mice were administered PSO orally at low (123 mg/day) or high (246 mg/day) doses via gavage, either for 14 days (short‐term) or 28 days (long‐term). COPD was induced by intraperitoneal injection of CSE once weekly for four consecutive weeks. Lung histopathology was evaluated using hematoxylin and eosin staining, and alveolar enlargement was quantified by measuring the mean linear intercepts (MLI). Bronchoalveolar lavage fluid (BALF) was collected to determine total and differential white blood cell counts. Oxidative stress was assessed by measuring total antioxidant activity and malondialdehyde (MDA) levels as indicators of lipid peroxidation. CSE exposure resulted in marked airspace enlargement, increased MLI values, and elevated macrophage and eosinophil counts in BALF. PSO treatment, particularly short‐term administration at a low dose, significantly reduced MLI values and attenuated overall inflammatory cell infiltration. Notably, PSO consistently decreased eosinophil counts across both short‐ and long‐term treatment groups. Furthermore, long‐term PSO administration led to a significant reduction in serum MDA levels, indicating enhanced antioxidant activity. PSO exhibited protective effects against CSE‐induced pulmonary inflammation, alveolar destruction, and oxidative stress in a murine COPD model. These findings support the potential role as a dietary supplement for the prevention or attenuation of COPD progression.

## Graphical abstract

PSO exhibited protective effects against CSE‐induced pulmonary inflammation by reducing macrophage and eosinophil infiltration, preventing alveolar destruction, and enhancing antioxidant defense by lowering serum MDA levels in a murine COPD model. These findings support the potential role as a dietary supplement for the prevention or attenuation of COPD progression.

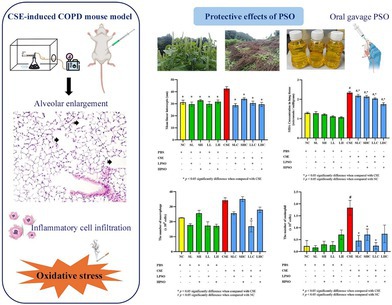

## 1 Introduction

Chronic obstructive pulmonary disease (COPD) is a progressive and long‐term inflammatory lung condition characterized by alveolar destruction, enlargement, and irreversible airflow limitation, which collectively impair normal respiratory function. As of 2021, COPD is the third leading cause of mortality globally and is projected to become the leading cause of death by 2030 (Pizzini et al. 2018). Tobacco use is the primary etiological factor in the development of COPD. It induces pulmonary inflammation, oxidative stress, and systemic effects, including comorbid diseases such as cardiovascular disease, skeletal muscle atrophy, and osteoporosis. Cigarette smoke contains high levels of free radicals, which contribute to oxidative stress and promote inflammatory responses within the lungs. This stress leads to the secretion of multiple cytokines, including TGF‐β and FGFs, which collectively stimulate fibrotic remodeling of small airways. In addition, the inflammatory processes involve the release of circulating immune cells via chemokines, and the release of pro‐inflammatory cytokines such as TNF‐α, IL‐1β, IL‐6, and IL‐8, further exacerbating pulmonary inflammation (Hikichi et al. 2019). The clinical manifestations of COPD, particularly airway obstruction and emphysema, are driven by these inflammatory and fibrotic mechanisms. Contemporary treatment strategies primarily aim to alleviate symptoms, improve quality of life, and prevent disease progression, rather than address the underlying pathology. However, prolonged use of pharmacological therapies can lead to adverse effects, including weight gain, glucose intolerance, osteoporosis, cataracts, cardiovascular complications, and an increased susceptibility to infections (McEvoy and Niewoehner 1997). In recent years, there has been growing interest in natural compounds due to their therapeutic potential and rich phytochemical profiles. Among them, *Perilla frutescens* , a traditional herbal remedy, has been used to treat a variety of ailments such as asthma, infections, nausea during pregnancy, and depression. Reports suggest that perilla seeds possess significant anti‐inflammatory and antioxidative properties (Dhyani et al. 2019). According to Techaniyom et al., PSO contains approximately 61% oil, with a high proportion of polyunsaturated fatty acids (PUFAs), including alpha‐linolenic acid. The oil also comprises linoleic acid (~19%) and oleic acid (~9.5%) (Techaniyom et al. 2024). Previous research by Chang et al. has demonstrated that administration of perilla oil reduces bronchoalveolar inflammation by decreasing the expression of Th1 cytokines (IFN‐γ and IL‐2) and pro‐inflammatory cytokines (TNF‐α, IL‐1, and IL‐6) in lung and airway tissues. More recently, Yuan et al. (2022) investigated the effects of Perilla leaves extract (PLE) on airway inflammation in a COPD model. The study demonstrated that PLE ameliorated COPD‐associated inflammation both in vivo and in vitro, partly by inhibiting the TLR4/Syk/PKC/NF‐κB p65 signal pathway, suggesting its potential therapeutic value. Despite the demonstrated anti‐inflammatory and antioxidative effects of perilla oil in models of asthma and other inflammatory diseases (Paradee et al. 2021; Chang et al. 2012; Deng et al. 2007; Lim et al. 2014), its role in COPD has not been adequately explored. In the present study, we aim to evaluate the protective effects of Thai *Perilla frutescens* seed oil (PSO) against lung inflammation and oxidative stress in a cigarette smoke extract (CSE)‐induced mouse model of COPD. The findings of this study may provide valuable insights into the potential of PSO as a functional food component or complementary therapeutic agent in the prevention or management of COPD.

## 2 Materials and Methods

### 2.1 *Perilla frutescens* Seed Oil (PSO) Material

*Perilla frutescens* seeds of Thai origin were obtained from a previous study conducted by Techaniyom et al. (2024). The seeds were cultivated in Mae Suai District, Chiang Rai, Thailand, and harvested between November and January. Following harvest, the seeds were dried and subjected to cold‐press extraction (temperature < 60°C) at the Institute of Nutrition, Mahidol University. The dosage was calculated based on body surface area (BSA) as recommended by Nair and Jacob (2016) using the body surface area to calculate the appropriated dosage of PSO (Nair and Jacob 2016). Accordingly, mice received either a low dose (0.132 mL/day) or a high dose (0.264 mL/day) of PSO.

### 2.2 Cigarette Smoke Extract (CSE) Preparation

Cigarette smoke extract (CSE) was prepared following the method described by Kettawan et al. (2024). The extraction apparatus consisted of L‐shaped glass tubing connected to a volumetric flask placed within a sealed plastic chamber containing phosphate‐buffered saline (PBS). One filtered Marlboro cigarette (Marlboro, Philip Morris, New York, NY, USA) was attached to the glass tube and ignited. The smoke generated was drawn through 1 mL of PBS using a vacuum pump at a constant pressure. The pH of the resulting solution was adjusted to 5.2–5.4. This CSE was then filtered through a 0.22 μM pore filter to remove particulate matter. Fresh CSE solution was prepared for each intraperitoneal injection.

### 2.3 Animal Procedure

All animal protocols were reviewed and approved by the Siriraj Animal Care and Use Committee (SI‐ACUC), Faculty of Medicine Siriraj Hospital, Mahidol University (SI‐ACUP no. 001/2565). Eight‐week‐old male C57BL/6J mice (20–23 g) were purchased from Nomura Siam International Co. Ltd. Mice were maintained under standard laboratory conditions (12‐h light/dark cycle, temperature 23°C ± 2°C) with ad libitum access to food and water. At the end of the experimental period, mice were euthanized by 100% CO_2_ inhalation followed by cervical dislocation. Death was confirmed by the absence of reflexive hind limb movement and urinary incontinence. Blood samples were collected via cardiac puncture. Subsequently, bronchoalveolar lavage fluid (BALF) and lung tissues were harvested. Lungs were flushed with normal saline, and BALF was collected using a 22‐gauge intravenous catheter (Terumo, Leuven, Belgium). For histological and biochemical analyses, lung tissues were inflated with 0.4% Ultrapure low‐melting point agarose (Invitrogen, Carlsbad, CA, USA) in sterile normal saline solution (NSS) at a transpulmonary pressure of 25 cmH_2_O, monitored using a Traceable manometer (Thermo Fisher Scientific Inc., MA, USA).

### 2.4 Experimental Design

**FIGURE 1:**
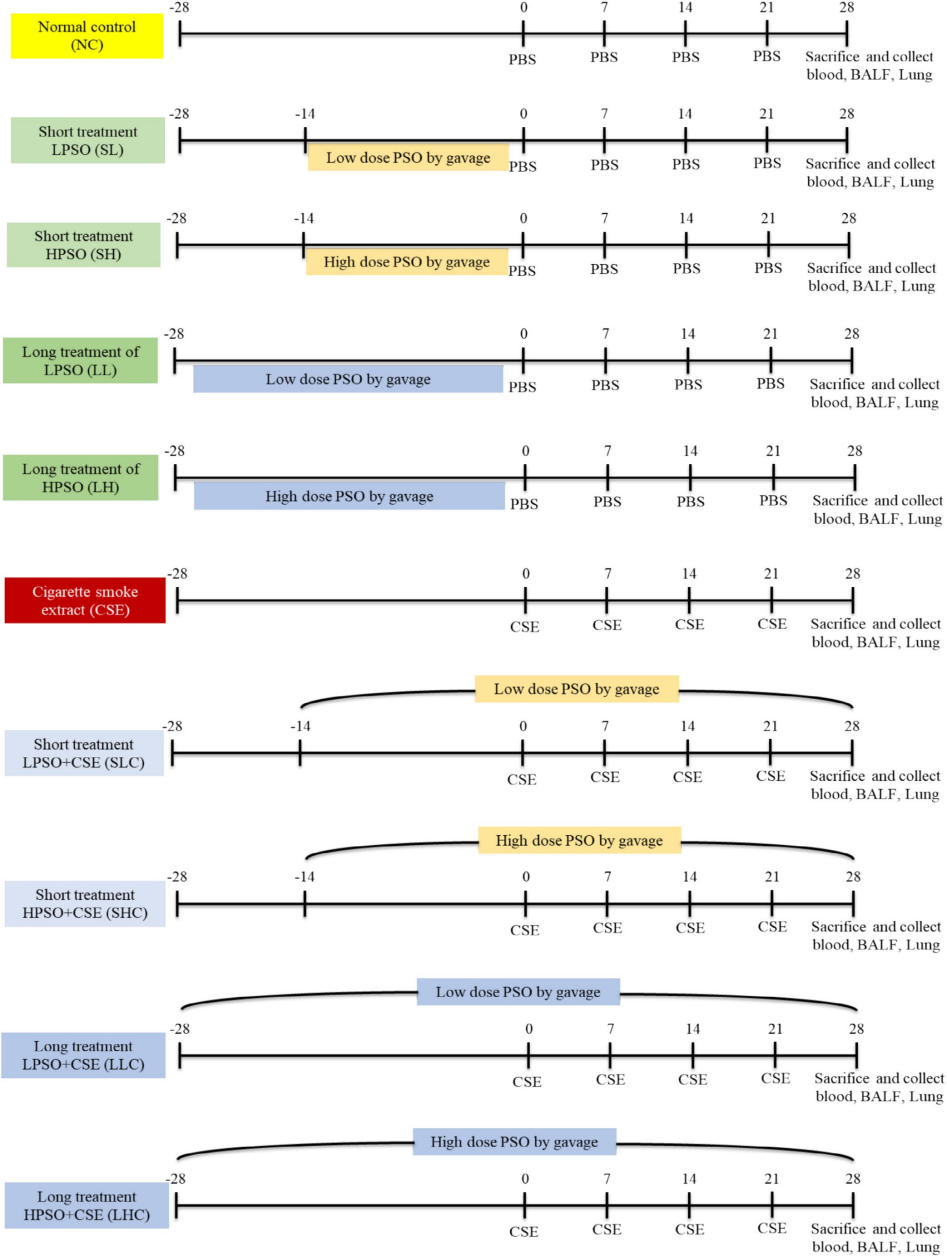
Schematic representation of the experimental protocol. BALF, bronchoalveolar lavage fluid; CSE, cigarette smoke extract; i.p., intraperitoneal injection; LH, long treatment of high dose PSO; LHC, long treatment of high dose PSO and CSE‐induced; LL, long treatment of low dose PSO; LLC, long treatment of low dose PSO and CSE‐induced; PBS, phosphate buffer saline; PSO, perilla seed oil; SH, short treatment of high dose PSO; SHC, short treatment of high dose PSO and CSE‐induced; SL, short treatment of low dose PSO; SLC, short treatment of low dose PSO and CSE‐induced.

Mice were randomly divided into 10 groups, with 6 mice per cage. They were administered either a low or high dose of PSO daily for either a short‐term (14 days) or long‐term (28 days) period prior to the induction of COPD. PSO was administered orally by gavage. At 8 weeks of age (designated as Day 0), mice in the COPD groups were intraperitoneally injected with 0.5 mL of freshly prepared CSE on Days 0, 7, 14, and 21. Control mice received an equivalent volume of PBS on the same schedule. Throughout the study, mice in both control and COPD groups continued to receive either low or high doses of PSO via oral gavage, according to the designated treatment timeline (Figure 1).

### 2.5 Lung Histopathological Examination

Lung tissues were fixed in 10% formalin, sectioned midsagittal, embedded in paraffin, and subsequently sliced and stained with hematoxylin and eosin (H&E) following standard histological protocol. Whole lung sections were visualized using Photoshop CS5 (version 12.0) and imaged at 20× magnification using an Olympus BX43 light microscope (Olympus Corporation, Tokyo, Japan). Mean Linear Intercept (MLI) was assessed to quantify emphysematous changes and lung damage caused by smoke or particulate matter exposure. For detailed morphological analysis, H&E‐stained lung sections were also imaged at 200X magnification using an Olympus BX46 clinical microscope equipped with a mosaic imaging program (Olympus Inc., Tokyo, Japan). Grid lines were drawn vertically and horizontally across the whole‐lung section images using Photoshop CS5, dividing each image into 207 equal fields. From these, five central fields were randomly selected for MLI analysis. Each selected field was further subdivided into four areas (designated as areas 1, 2, 3, and 4), each measuring approximately 264.96 μm in both width and height. Two diagonally opposite areas (either areas 1 and 4 or 2 and 3) were chosen for quantifying alveolar intercepts, resulting in a total of 10 analysis areas per lung section. For MLI assessment, a grid consisting of 9 vertical and horizontal lines (10 × 10 small squares and 18 gridlines per area) was superimposed on each image. Alveolar wall interceptions were counted at each gridline crossing, yielding a total of 180 gridline crossings per area. The total number of alveolar intercepts from the 10 selected areas was used to calculate the MLI and assess airspace enlargement, as described by Alzoubi et al. (2018).

### 2.6 Total and Differential Cell Count in Bronchoalveolar Lavage Fluid (BALF)

#### 2.6.1 Total Cell Count

A 100 μL aliquot of BALF was mixed with an equal volume (100 μL) of 0.4% trypan blue (Thermo Fisher Scientific Inc., Waltham, MA, USA), resulting in a 1:2 dilution. The mixture was loaded into a Bright‐Line hemocytometer (Hausser Scientific, Horsham, PA, USA) and examined under a light microscope at 40× magnification. Cells in the four large corner squares of the hemocytometer were manually counted to determine the total cell number.

#### 2.6.2 Differential Cell Counting

Three hundred microliters of BALF were centrifuged at 600 rpm for 10 min in a cytocentrifuge chamber (Thermo Fisher Scientific Inc., Waltham, MA, USA). The resulting cell pellet was fixed with methanol and stained sequentially with eosin and 0.5% methylene blue for 4 min. Differential cell counts were performed using a light microscope at 100× magnification. The percentages of alveolar macrophages, neutrophils, eosinophils, and lymphocytes were determined based on morphological characteristics. Differential cell counts in BALF were conducted based on standard morphological characteristics under high‐power magnification, with representative cell types documented in Supporting Information Report 2.

### 2.7 Determination of TNF‐α Level in Serum

The whole blood was centrifuged at 4°C for 10 min to isolate the serum. TNF‐α levels were quantified using a commercial sandwich enzyme‐linked immunosorbent assay (ELISA) kit (Boster Biological Technology, Pleasanton, CA, USA), following the manufacturer's instructions.

### 2.8 Oxygen Radical Absorbance Capacity (ORAC) Analysis

To evaluate antioxidant capacity in COPD‐induced mice, both serum and BALF samples were analyzed using the ORAC assay. BALF samples were centrifuged at 1500 rpm for 60 min, while blood samples were centrifuged at 3500 rpm for 20 min at 4°C. A 500 μL aliquot of the supernatant was mixed with 3 mL of fluorescein working solution and incubated in a 37°C water bath. Subsequently, 500 μL of 2,2′‐azobis (2‐amidinopropane) di‐hydrochloride (AAPH) (Sigma‐Aldrich Pte. Ltd., Singapore) was added to initiate the oxidative reaction. Trolox (6‐hydroxy‐2,5,7,8‐tetramethylchroman‐2‐carboxylic acid) (Sigma‐Aldrich Pte. Ltd., Singapore) at concentrations ranging from 6.25 to 100 μM served as a standard. A blank control containing 500 μL of ORAC buffer working solution was included. Fluorescence was measured at an excitation wavelength of 493 nm and an emission wavelength of 515 nm using a spectrofluorometer. The fluorescence intensity was recorded every minute until it plateaued or decreased to near zero. The net area under the curve (AUC) was calculated by subtracting the AUC of the blank from the sample AUC and was compared to the Trolox standard curve. ORAC values were expressed as μM Trolox equivalents per mL sample.

### 2.9 Thiobarbituric Acid Reactive Substances (TBARS) Analysis

Lung tissues were processed to evaluate oxidative stress via malondialdehyde (MDA) quantification. Following centrifugation at 4°C for 10 min, MDA levels were measured using a commercial TBARS ELISA kit (Boster Biological Technology, Pleasanton, CA, USA) according to the manufacturer's protocol.

### 2.10 Statistical Analysis

All data were first tested for normal distribution. Comparisons between two groups were performed using an unpaired (independent) Student's *T*‐test. To assess differences among multiple groups, one‐way analysis of variance (ANOVA) followed by Bonferroni's post hoc test was applied. Results are expressed as the mean ± standard error of the mean (SEM). A *p*‐value of less than 0.05 was considered statistically significant. All statistical analyses were performed using the GraphPad Prism program, version 10.1.2 (324). Furthermore, all experimental data were analyzed using One‐way ANOVA followed by Tukey's test. To ensure full statistical transparency, the corresponding *F*‐values, degrees of freedom, and exact *p*‐values for all comparisons are provided in Supporting Information Report 1 (ANOVA Table).

## 3 Results

### 3.1 The Dietary PSO Exhibited Minimal Effect on Body Weight in CSE‐Induced Mice

**FIGURE 2:**
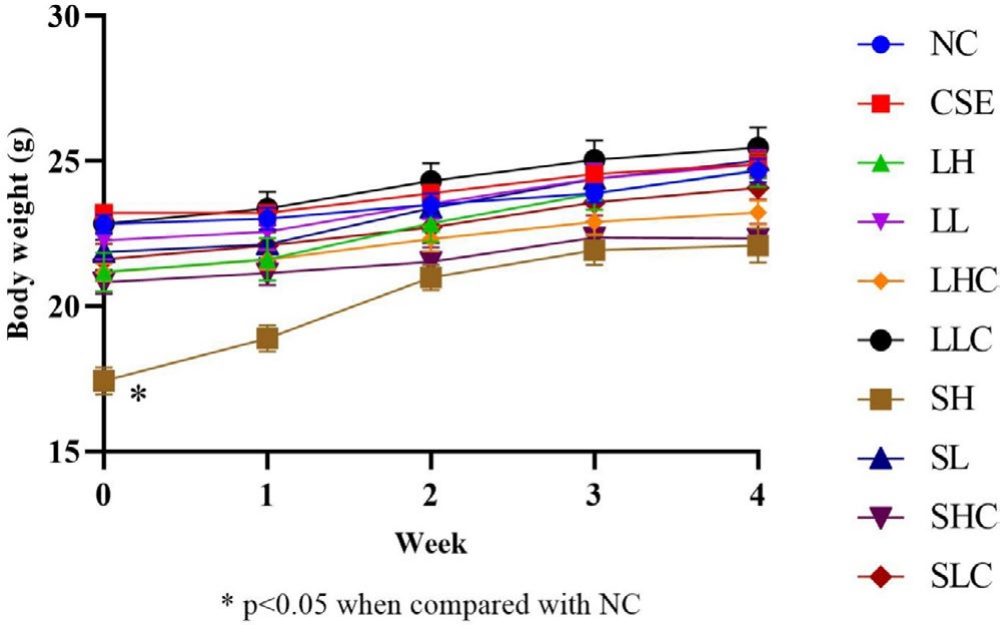
The body weight of mice on dietary PSO in CSE‐induced mice (g). Data were presented as mean ± SEM. *p < 0.05 (4 weeks).

Following the experimental protocol, mice received oral PSO supplementation once daily, and body weights were recorded daily until euthanasia. PSO was administered each morning, and differences in average body weights among the 10 groups were analyzed using two‐way ANOVA, with statistical significance set at *p* < 0.05 (Figure 2). Our results demonstrate that PSO administration, at both low and high doses, did not significantly influence the body weight of CSE‐induced mice throughout the experimental period. Body weight trajectories remained consistent across all experimental groups, including those receiving PBS or CSE alone. These findings indicate that PSO, at the administered dosages, does not adversely affect systemic growth or induce toxicity. This observation aligns with prior reports highlighting the biocompatibility and safety of edible plant oils in animal models. Although the SH group exhibited a lower baseline body weight at Day 0 compared to the NC group due to individual biological variation, there were no significant differences in the rate of body weight gain among any experimental groups throughout the study. This suggests that PSO intervention, even at high doses, does not adversely affect body weight or general health.

### 3.2 PSO Might Protect Against Possible Histopathological Alterations in CSE‐Induced Mice

**FIGURE 3:**
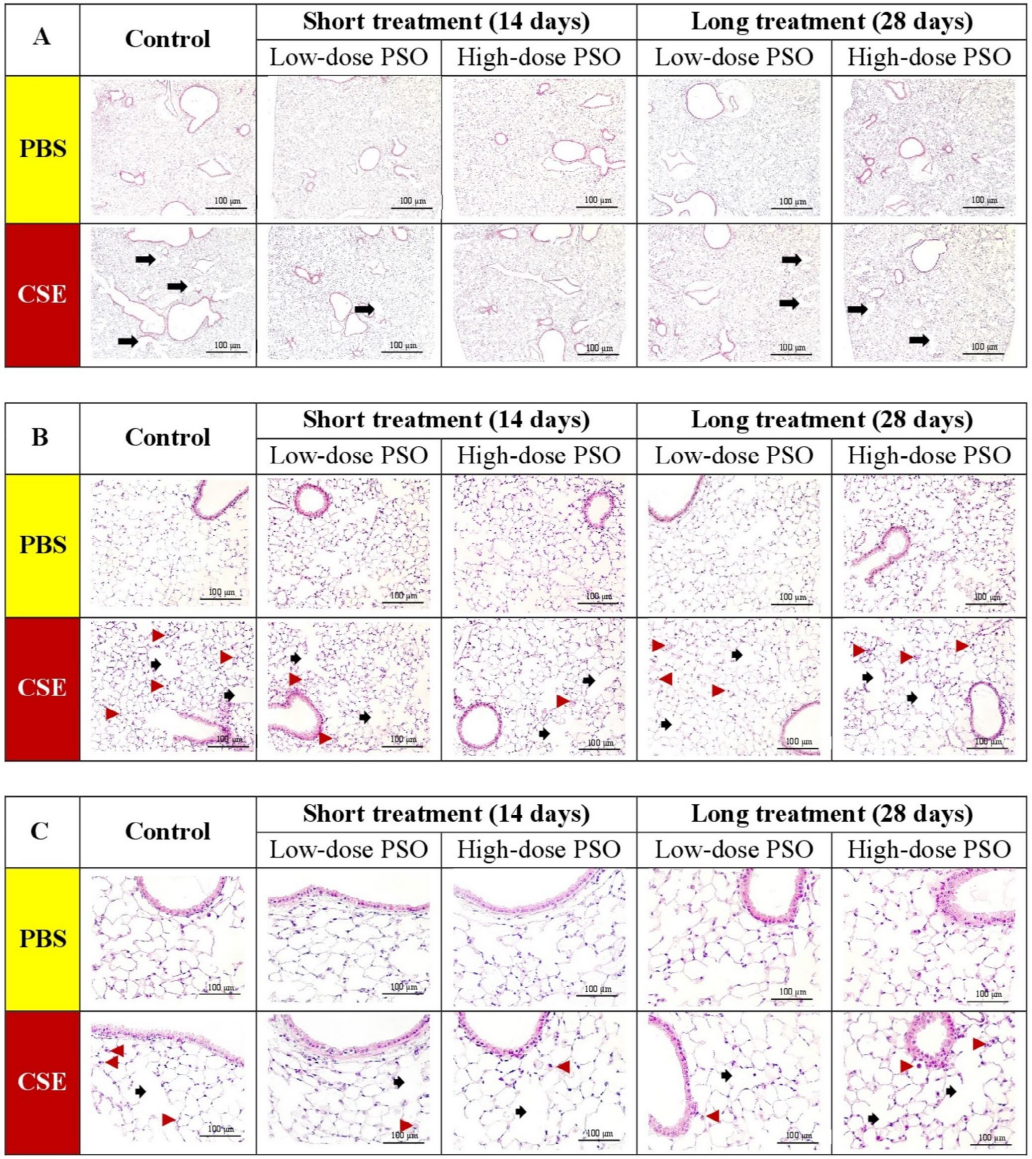
Representative histopathological images of lung tissue from mice following intraperitoneal injection of PBS and CSE at 5× (A), 20× (B), and 40× (C) magnification. The black arrow represents incomplete alveolar structure with enlargement of airspaces, and the red arrow represents infiltration of inflammatory cells.

Hematoxylin and eosin (H&E) staining of lung sections revealed distinct pathological alterations in the cigarette smoke extract (CSE)–induced COPD mouse model when compared with normal control mice. At lower magnification, CSE‐exposed lung tissues showed a loss of normal alveolar architecture with marked enlargement of airspaces, consistent with emphysematous remodeling. The alveolar septa appeared thinned and in some regions were partially destroyed, leading to the formation of irregular and enlarged alveolar spaces. These structural changes were reflected in increased mean linear intercept (MLI) values, confirming quantitative evidence of alveolar enlargement. At higher magnification, more detailed pathological features became evident. The alveolar walls displayed thinning and focal rupture, with infiltration of inflammatory cells within the alveolar and peri‐bronchial regions. Evidence of epithelial damage and localized congestion was also noted. Collectively, these findings indicate that CSE exposure induces severe alveolar injury, inflammatory cell recruitment, and progressive emphysematous changes, recapitulating key histopathological characteristics of COPD. In contrast, PSO‐treated groups subjected to CSE exposure exhibited notable improvements in lung parenchyma, including reduced alveolar destruction and airspace enlargement (Figure 3).

**FIGURE 4:**
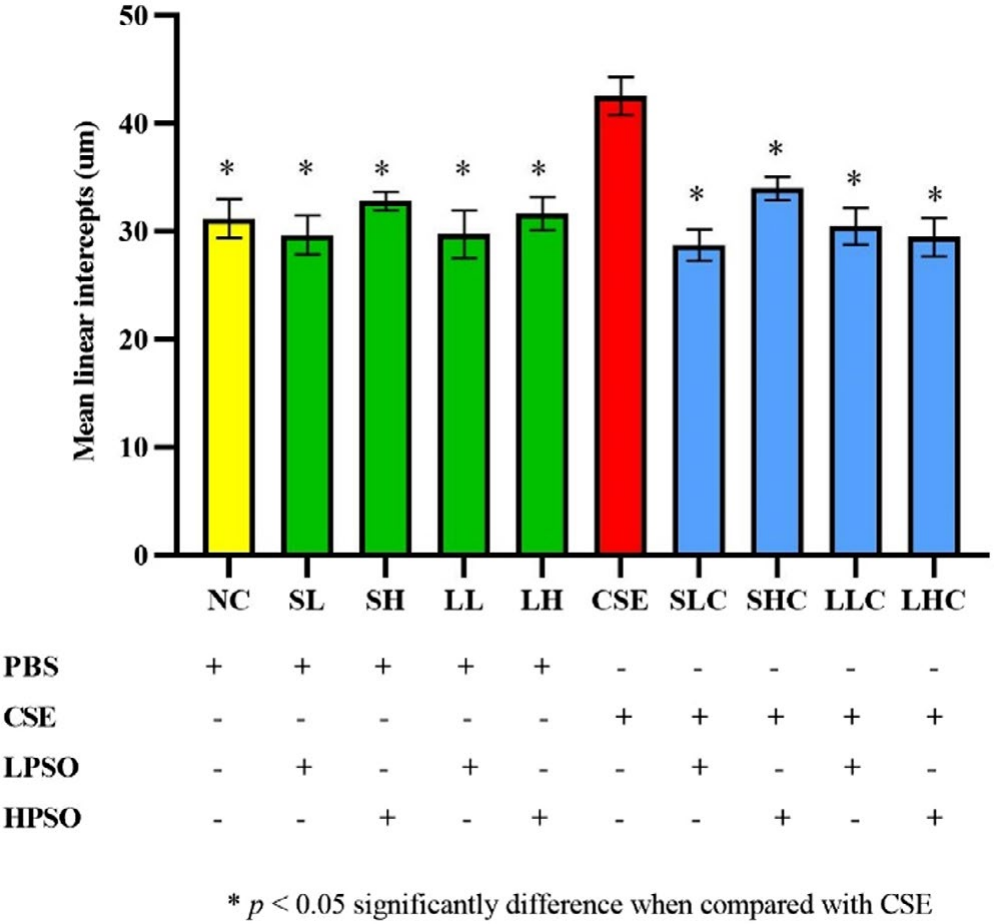
MLI measurements in C57BL/6J mice following intraperitoneal injection of CSE. Data are expressed as mean ± SEM. *p < 0.05 compared with the CSE group. CSE, cigarette smoke extract; HPSO, high dose of perilla seed oil; LPSO, low dose of perilla seed oil; PBS, phosphate buffered saline.

Quantitative analysis confirmed a significant reduction in MLI in PSO‐treated groups compared to the CSE‐only group, suggesting a protective effect of PSO against structural lung damage associated with COPD. Notably, this effect was more pronounced with long‐term treatment, regardless of PSO dose. MLI values were significantly increased in CSE‐induced mice compared to the normal control (NC) group (*p* < 0.05). However, administration of either low or high doses of PSO under short‐ and long‐term treatments resulted in MLI values that were not significantly different from NC. Importantly, both short‐ and long‐term PSO treatments significantly reduced MLI values in CSE‐exposed mice when compared to the untreated CSE group (*p* < 0.05) (Figure 4).

### 3.3 Anti‐Inflammatory Effects of PSO in CSE‐Induced COPD in Mice

**FIGURE 5:**
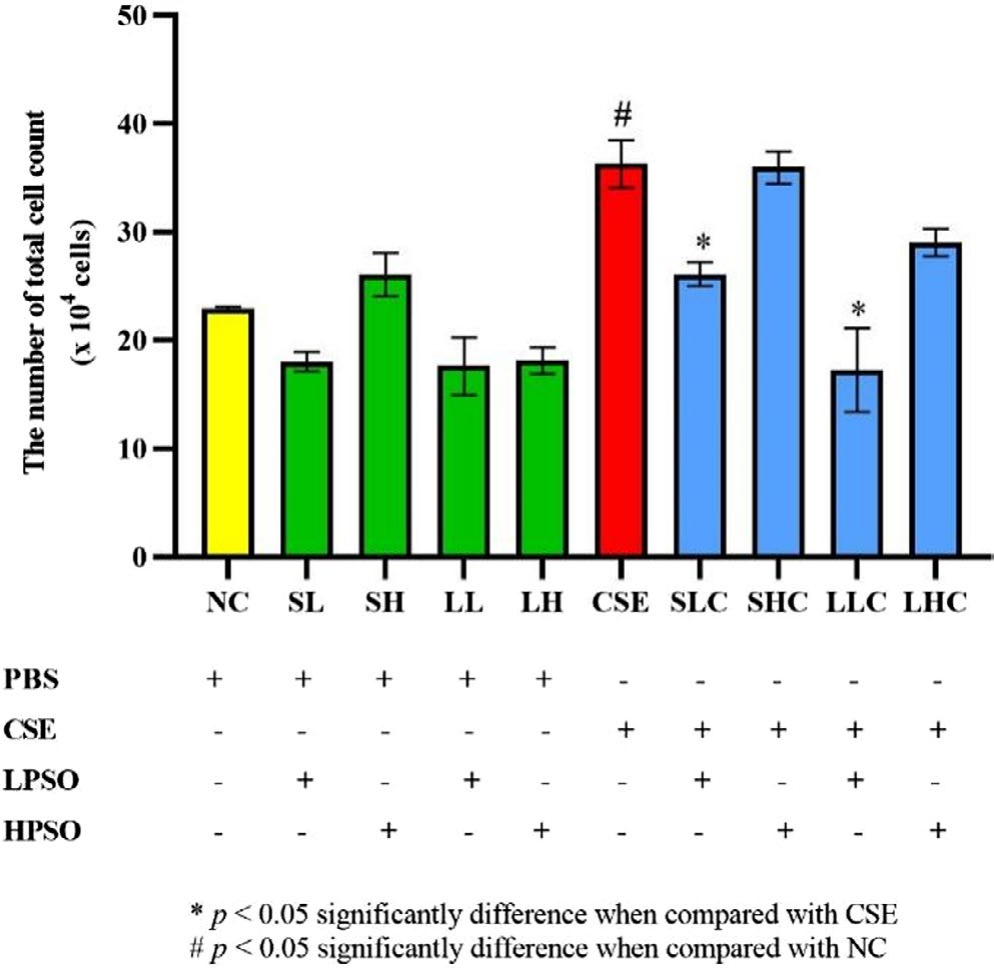
Total cells count white blood cells in BALF of C57BL/6J mice induced by intraperitoneal injection of CSE. Data are expressed as mean ± SEM. CSE, cigarette smoke extract; HPSO, high dose of perilla seed oil; LPSO, low dose of perilla seed oil.

**FIGURE 6:**
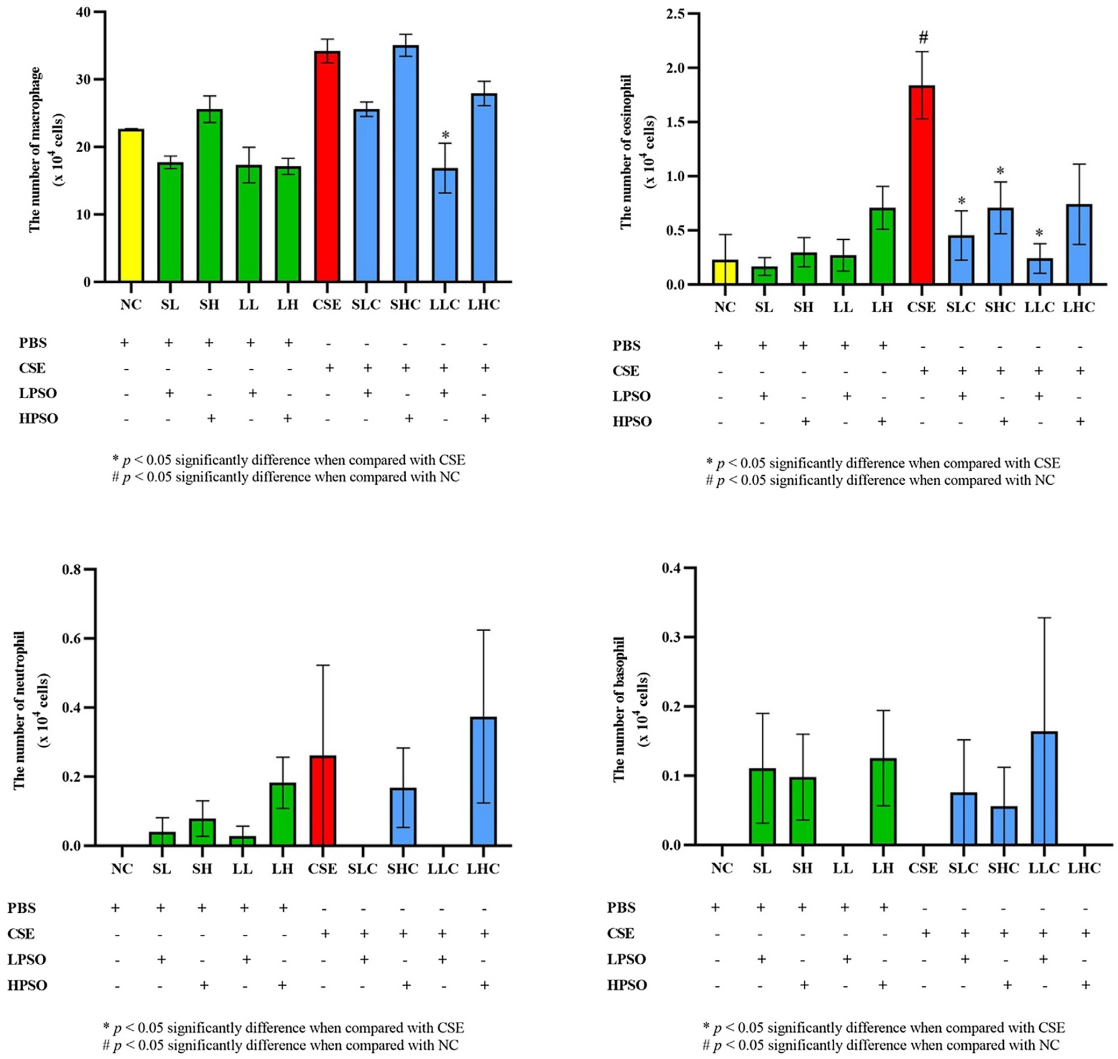
Differential cell count (×10^4^ cells) in BALF of C57BL/6J mice induced by intraperitoneal injection of CSE. Data were presented as mean ± SEM.

To evaluate the anti‐inflammatory potential of PSO, total inflammatory cell counts and differential cell profiles in bronchoalveolar lavage fluid (BALF) were assessed. PSO administration led to a significant reduction in total inflammatory cell counts in both SLC and LLC groups, indicating an attenuation of the CSE‐induced inflammatory response (Figure 5). Differential cell analysis revealed a marked reduction in macrophages and eosinophils, particularly in the LLC group, with statistically significant differences compared to the CSE‐ group. These findings suggest that long‐term PSO treatment may suppress macrophage and eosinophil infiltration—key contributors to COPD pathogenesis (Figure 6). In contrast, no significant changes were observed in neutrophil and basophil counts across groups, suggesting that PSO may exert selective immunomodulatory effects rather than broad‐spectrum immune suppression.

### 3.4 Effect of PSO on the Levels of TNF‐α in Serum of CSE‐Induced COPD in Mice

Serum levels of TNF‐α were quantified using ELISA. TNF‐α levels were undetectable across all experimental groups, including the CSE‐exposed and PSO‐treated mice. This may be attributed to the assay's sensitivity threshold or the timing of sample collection relative to cytokine release dynamics. Although TNF‐α levels were not detected, the observed improvements in lung histology and reduction in inflammatory cell infiltration suggest that PSO may exert systemic and pulmonary anti‐inflammatory effects, potentially mediated through mechanisms independent of serum TNF‐α modulation.

### 3.5 Effect of PSO on Antioxidant Activity in Serum and BALF of CSE‐ Induced COPD in Mice

The ORAC assay revealed that PSO treatment significantly enhanced the total antioxidant capacity in both serum and BALF of treated mice, counteracting the oxidative stress induced by CSE.

**FIGURE 7:**
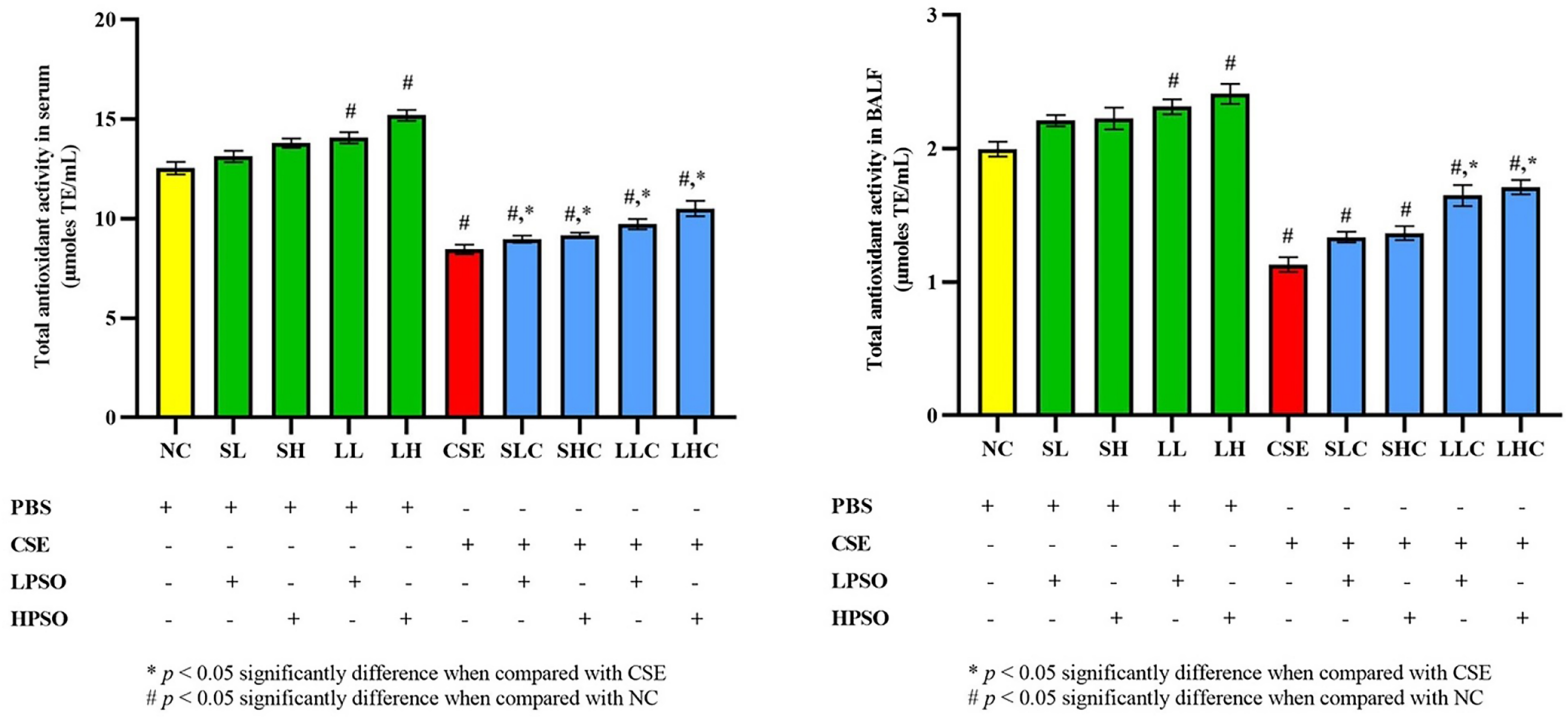
Total antioxidant activity in serum and BALF of CSE‐induced COPD in mice. All data presented as mean ± SEM. CSE, cigarette smoke extract; HPSO, high dose of perilla seed oil; LPSO, low dose of perilla seed oil.

*In serum*, antioxidant activity was assessed using ORAC assay. Exposure to CSE resulted in a marked reduction in total antioxidant capacity compared to the NC group (*p* < 0.0001). However, administration of PSO led to a significant increase in antioxidant capacity across both short and long‐term treatments at both low and high doses (SLC, SHC LLC and LHC; *p* < 0.0001 for all groups). Notably, even in the absence of CSE exposure, long‐term administration of PSO alone at both low and high doses significantly increased serum antioxidant capacity compared to the NC group (LL; *p* = 0.0083 and LH; *p* < 0.0001) (Figure 7).

Similarly, in BALF, the ORAC assay demonstrated a significant reduction in antioxidant capacity in the CSE‐exposed group compared to NC (*p* < 0.0001). PSO supplementation significantly restored antioxidant levels in the long‐term low‐dose and high‐dose treatment groups (LLC and LHC; *p* < 0.0001). This marked improvement highlights the potent antioxidant effect of PSO, particularly in long‐term administration (Figure 7).

### 3.6 Effect of PSO on Malondialdehyde (MDA) Levels in Lung Tissue of CSE‐ Induced COPD in Mice

**FIGURE 8:**
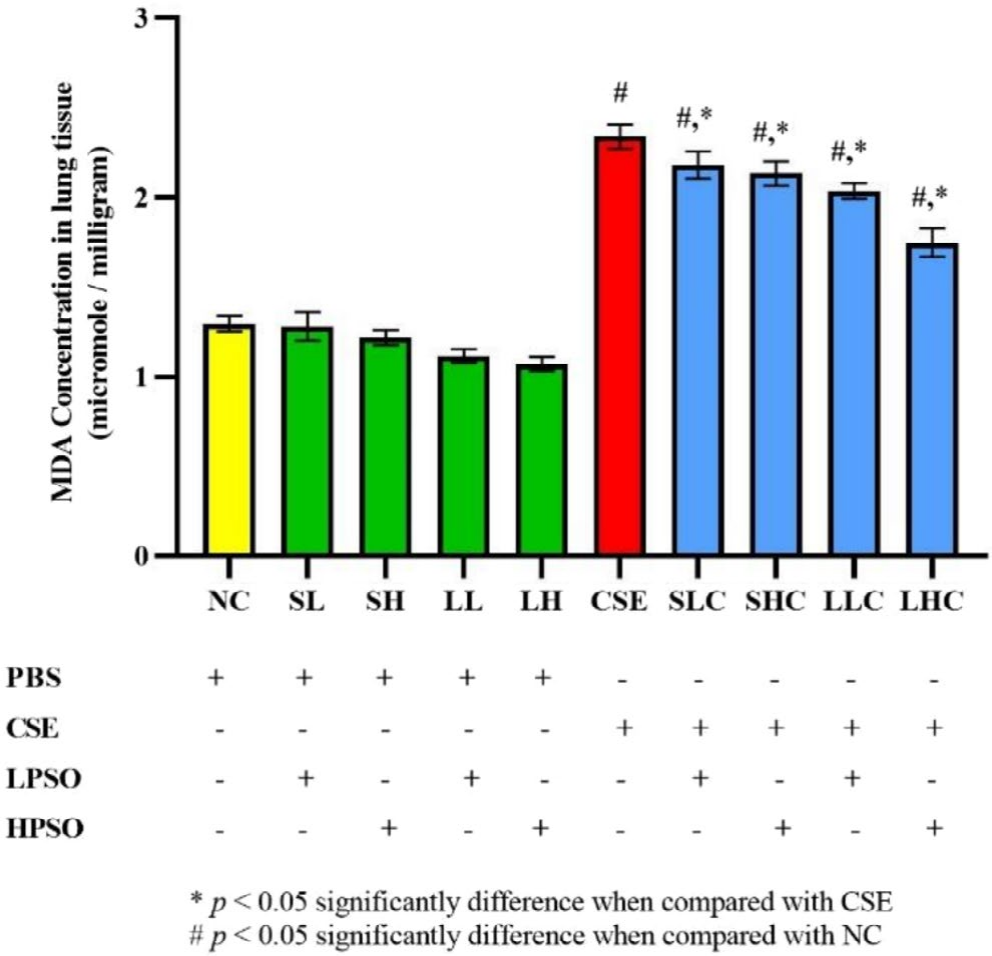
The level of MDA in lung tissue of CSE‐induced COPD in mice. All data presented as mean ± SEM. CSE, cigarette smoke extract; HPSO, high dose of perilla seed oil; LPSO, low dose of perilla seed oil.

Lipid peroxidation in lung tissue was evaluated using the thiobarbituric acid reactive substances (TBARS) assay. CSE exposure significantly increased MDA levels, indicating increased lipid peroxidation and oxidative damage in lung tissues (*p* < 0.0001 vs. NC). PSO treatment significantly reduced MDA levels in all treatment groups, including both short‐term and long‐term administrations at low and high doses (SLC, SHC LLC, and LHC; *p* ≤ 0.0001) (Figure 8). The most pronounced reduction in MDA levels was observed in long‐term treatment groups, underscoring the sustained antioxidant and protective effects of PSO. These findings support the role of PSO in mitigating oxidative stress and preserving lung tissue integrity through its lipid peroxidation‐inhibiting properties.

## 4 Discussion

Chronic obstructive pulmonary disease (COPD) is a progressive inflammatory lung disorder characterized by persistent airflow limitation, alveolar wall destruction, and enlargement of airspaces. Globally, COPD remains a significant public health challenge, ranking as the third leading cause of death worldwide in 2021—a figure projected to increase further by 2030 (Pizzini et al. 2018). The clinical manifestations of COPD, such as airway obstruction and emphysematous changes, are multifactorial in origin. Among these, cigarette smoking is the most prominent etiological factor and a major risk for disease onset and progression. In experimental models, alveolar enlargement is a fundamental feature of emphysema and is commonly quantified using the mean linear intercept (MLI) method (Brusselle et al. 2006). In the present study, emphysema was induced in mice via intraperitoneal injection of CSE, resulting in significant alveolar destruction and airspace enlargement. This was accompanied by elevated recruitment of white blood cells in BALF, indicating a robust inflammatory response. Our findings showed a significant increase in MLI in the lungs of CSE‐treated mice, confirming the development of emphysematous changes. These findings align with those of Kettawan et al. (2024), who reported similar pathological alterations following intraperitoneal administration of CSE in mice, including enlarged alveolar spaces and thinning of alveolar walls. Furthermore, He et al. (2015) demonstrated that both cigarette smoke (CS) inhalation and intraperitoneal CSE injection effectively induced emphysema in mice. They observed comparable pathological features between the two models, such as impaired lung function, alveolar wall damage, increased apoptosis of alveolar septal cells, and increased inflammatory cell infiltration in BALF. Similarly, Li et al. (2020) reported that CSE induces lung epithelial cell death by decreasing PRMT6 at both mRNA and protein levels. This reduction subsequently increases PI3Kp85 expression and stabilizes PTEN, which, along with decreased PDK1, ultimately results in reduced AKT phosphorylation—highlighting a mechanistic pathway contributing to CSE‐induced lung damage. Given these consistent outcomes, CSE was selected for the current study due to its comparable efficacy with CS in inducing emphysema, while offering practical advantages such as reduced exposure risk for researchers and simplified experimental logistics. Although this study provides robust evidence of PSO's protective effects through MLI quantification and BALF inflammatory cell analysis, specific lung‐function tests—such as airway resistance and lung compliance—were not performed. Future studies incorporating these physiological measurements would further elucidate the functional impact of PSO on respiratory mechanics. Furthermore, in this study, COPD was induced via intraperitoneal injection of CSE, which successfully recapitulated key features such as alveolar injury and inflammatory cell recruitment. However, this model may not fully mimic the long‐term physiological impact or the classical markers associated with chronic inhalation exposure. This should be considered when interpreting the systemic versus localized effects of PSO.

This study also evaluated the protective effect of PSO, which is rich in alpha‐linolenic acid in a CSE‐induced COPD mouse model. PSO was administered via oral gavage at both low (123 mg/day) and high (246 mg/day) doses over short (14‐day) and long (28‐day) periods. The PSO‐only group served to establish the baseline safety and physiological impact of the oil. Our data indicated that PSO administration alone did not significantly alter lung morphology (MLI) or inflammatory cell profiles compared to the normal control, regardless of the duration. This confirms that the protective effects observed in the CSE + PSO groups are specifically due to the intervention against CSE‐induced pathology. Dosage calculations were performed based on body surface area, following the methodology described by Nair et al., ensuring translational relevance and clinical safety (Nair and Jacob 2016). The dose conversion also considered dietary recommendations from the Thai Bureau of Nutrition, which suggest a daily intake of approximately 30 g of oil for a 60 kg adult. Throughout the treatment period, there were no significant changes in body weight among PSO‐treated mice, indicating that the observed protective effects were not confounded by systemic metabolic alterations. These findings support the potential of PSO as a functional dietary component or adjunctive nutritional strategy for managing chronic lung inflammation and emphysema.

The protective effects of PSO were reflected in the significant reduction of MLI values in treated mice, suggesting that PSO helped preserve alveolar integrity. Perilla seeds are known for their potent anti‐inflammatory and antioxidant properties (Dhyani et al. 2019). PSO is particularly rich in polyunsaturated fatty acids (PUFAs), especially in alpha‐linolenic acid (ALA), which constitutes approximately 61% of its composition. ALA serves as a metabolic precursor for the essential omega‐3 fatty acids EPA and DHA, which are well recognized for their anti‐inflammatory and tissue‐protective roles (Asif 2011). Additionally, previous studies have demonstrated the therapeutic potential of Perilla‐derived compounds in respiratory inflammation. For instance, Yuan et al. (2022) investigated the effect of perilla leaf extract (PLE) on COPD‐associated airway inflammation and found that it significantly attenuated inflammation both in vitro and in vivo. The underlying mechanism was attributed to suppression of the TLR4/Syk/PKC/NF‐κB p65 signaling pathway, further supporting the anti‐inflammatory potential of perilla‐derived bioactive compounds in pulmonary disease.

Cigarette smoke extract induces pulmonary inflammation primarily by continuously recruiting inflammatory cells, notably macrophages and eosinophils, into lung tissue. These immune cells play pivotal roles in the inflammatory cascade associated with chronic obstructive pulmonary disease (COPD). Consistent with previous study, Xu et al. (2023) reported that COPD patients with elevated peripheral blood eosinophil counts exhibit more pronounced declines in lung function and more severe emphysematous changes. Similarly, in a recent research it was observed that increased eosinophil infiltration in the lungs correlates with altered airway microbiota, suggesting a potential link between eosinophilic inflammation and microbial dysbiosis in COPD (Higham et al. 2024). Furthermore, Çolak et al. (2024) demonstrated that elevated blood eosinophil levels are associated with an accelerated decline in lung function, underscoring the role of type 2 inflammation in the progressive pathogenesis of COPD. Lee et al. (2024) further elucidated the pathogenic role of eosinophils, showing that these cells exacerbate airway remodeling and inflammation through the secretion of cytotoxic granules and pro‐inflammatory cytokines. In the present study, PSO demonstrated protective effects in a mouse model of COPD, particularly by attenuating airspace enlargement. Notably, high‐dose PSO significantly reduced eosinophilic infiltration and macrophage accumulation in BALF, suggesting an immunomodulatory effect of PSO in mitigating the inflammatory response induced by CSE exposure. Moreover, PSO administration markedly reduced MDA levels—a biomarker of lipid peroxidation and oxidative tissue injury—while enhancing total antioxidant capacity in both serum and BALF. These findings indicate a potent antioxidant effect of PSO in counteracting oxidative damage associated with CSE exposure. The anti‐inflammatory and antioxidant effects of PSO are likely mediated by its high content of PUFAs, particularly ALA, a precursor to EPA and DHA—key omega‐3 fatty acids, both of which are well‐recognized for their immunoregulatory and cytoprotective properties (Asif 2011; Bodur et al. 2025). The *Perilla frutescens* seed oil (PSO) used was sourced from the Royal Project Foundation (Thailand). The chemical composition of this specific oil batch has been fully characterized (Techaniyom et al. 2024). This previous analysis confirmed a standardized profile with alpha‐linolenic acid (ALA) at 60.10% and significant phenolic content, ensuring batch reproducibility for the current study. Furthermore, it is important to consider that while ALA is the primary active component of PSO (comprising up to 60%), the oil remains a complex biological mixture containing other fatty acids, such as linoleic and oleic acids, alongside various phytochemicals. The lack of a strict dose‐dependent effect in certain outcomes may be attributed to the complex interactions among these constituents within the CSE‐induced COPD model, which could influence the overall therapeutic efficacy in a non‐linear manner.

Interestingly, while PSO exhibited beneficial effects at both low and high doses, the high‐dose treatment did not consistently yield superior outcomes. In some cases, it was associated with diminished or even adverse effects. Several mechanistic explanations may account for this dose‐dependent variability. Firstly, although PSO is rich in antioxidants like ALA, excessive PUFAs intake can paradoxically increase oxidative stress due to the inherent susceptibility of omega‐3 fatty acids to lipid peroxidation. This process generates reactive aldehydes such as MDA, which can compromise membrane integrity and amplify inflammatory signaling if not efficiently neutralized by endogenous antioxidant defenses (Yin et al. 2011; Halliwell and Gutteridge 2015). Second, chronic administration of high amounts of dietary oil may disrupt lipid metabolism and energy homeostasis in mice. Even when derived from health‐promoting sources, excess fat intake can impair liver function, alter gut microbiota composition, or promote ectopic lipid deposition, thereby triggering systemic inflammation and metabolic dysregulation (Caesar et al. 2015). Third, while omega‐3 PUFAs are known to modulate immune function, excessive intake may lead to immune suppression or unfavorable shifts in immune cell polarization. For instance, high doses may inhibit macrophage activity or excessively suppress T‐cell responses, potentially compromising host defense and immune homeostasis (Calder 2017; Shaikh and Edidin 2006). Fourth, dietary oils and natural bioactive compounds often exhibit biphasic or hormetic dose–response behavior, wherein low doses elicit beneficial effects, but higher doses result in reduced efficacy or toxicity. This non‐linear phenomenon is well‐documented in nutraceutical research (Calabrese 2004; Mattson 2008). Finally, high levels of exogenous antioxidants may disrupt physiological redox signaling pathways, such as the nuclear factor erythroid 2‐related factor 2 (Nrf2) pathway, which regulates endogenous antioxidant gene expression. Over‐supplementation may blunt adaptive stress responses and reduce long‐term resilience of lung tissue to oxidative insults (Dinkova‐Kostova and Abramov 2015; Steinhubl 2008).

To contextualize these findings for human application, the equivalent human dose (HED) of PSO was calculated based on animal study, in which 20 g mice received a low dose of 0.132 mL/day. The resulting HED is approximately 0.535 mL/kg, corresponding to 32.1 mL (or 29.5 g) of PSO per day for a 60 kg adult. According to the World Health Organization (WHO), total fat intake should not exceed 30% of daily energy intake, which equals ~66.7 g of fat for a 2000 kcal/day diet (Who and FAO Expert Consultation 2003). Therefore, 29.5 g of PSO falls within this recommended range. Additionally, WHO guidelines recommend that 6%–11% of total energy intake be derived from PUFAs (approximately 13–24 g/day). Although the PSO does slightly exceed this range, it remains within acceptable limits, particularly considering the known health benefits of omega‐3 fatty acids. Nonetheless, it is important to consider total dietary fat intake from all sources to ensure nutritional balance and safety.

In conclusion, given that current pharmacological treatments for COPD largely provide symptomatic relief without altering disease progression, dietary interventions such as PSO may represent a promising adjunctive strategy for mitigating lung inflammation and oxidative damage in the early or moderate stages of COPD. CSE contains high levels of free radical molecules with unpaired electrons—which promote COPD pathogenesis via oxidative stress and chronic inflammation. PSO, with its potent antioxidant capacity, may neutralize these radicals, stabilize molecular targets and attenuate inflammatory pathways. Further mechanistic studies and well‐controlled clinical trials are warranted to validate these findings and to elucidate the molecular underpinnings of PSO's therapeutic potential in COPD management.

## 5 Conclusion

This study demonstrates that dietary supplementation with PSO, a rich source of ALA, confers significant protective effects against the pathological features of COPD in a CSE‐induced mouse model. Administration of PSO effectively attenuated airspace enlargement, preserved alveolar architecture, and significantly reduced both eosinophilic and total inflammatory cell infiltration in lung tissues. These beneficial effects are likely mediated through the oil's robust anti‐inflammatory and antioxidant properties. Notably, both low and high doses of PSO were well tolerated in vivo, with no indications of systemic toxicity or adverse effects. These findings suggest that PSO may serve as a promising functional food component or adjunct dietary intervention to support pulmonary health, particularly among individuals at risk for or diagnosed with chronic respiratory conditions such as COPD. However, translational relevance requires confirmation through human clinical trials. Further investigation into the underlying molecular pathways, including those related to redox homeostasis and immune modulation, is warranted to fully elucidate PSO's therapeutic potential. In this study, the Supporting Information include Supporting Information Report 1: ANOVA table and Supporting Information Report 2: Morphological analysis of cell counts in BALF.

## 6 Implications and Limitations

The results of this study underscore the potential of PSO as a multi‐targeted therapeutic agent in mitigating the development and progression of COPD. The protective effects observed—ranging from attenuation of alveolar damage to modulation of inflammatory and oxidative stress responses—suggest that PSO acts through multiple, potentially synergistic mechanisms. This positions PSO as a viable candidate for integrative approaches to COPD management, especially in settings where conventional therapies are limited or patients seek nutritional adjuncts to pharmacological treatments.

Nonetheless, several limitations must be acknowledged. First, the absence of measurable levels of TNF‐α constrains our ability to evaluate systemic pro‐inflammatory cytokine dynamics. In this study, the inability to detect certain serum cytokines may be attributed to two factors: the one‐week interval between the final CSE challenge and blood collection, which may have missed the peak systemic inflammatory response, and the limited serum volume (approx. 200 μL) obtained. This volume was prioritized for oxidative stress analysis (ORAC and MDA) to evaluate the core antioxidant mechanisms of PSO. Given that pro‐inflammatory cytokines like TNF‐α often exhibit rapid and transient elevation following exposure, the one‐week interval prior to blood collection may have missed the peak circulating levels. The current study demonstrates the protective role of PSO through histopathological and cellular analyses; molecular assessments—such as quantitative PCR (qPCR) to examine the mRNA expression of key inflammatory cytokines and chemokines in lung tissue—were not performed. This represents an important direction for future mechanistic studies to further define the molecular pathways involved in PSO's therapeutic effects.

Second, the study did not include molecular analyses of specific oxidative stress markers or expression levels of antioxidant enzymes and pro‐inflammatory mediators, which would be instrumental in delineating the mechanistic pathways of PSO action. Furthermore, while no adverse effects on body weight were observed, this study did not include a comprehensive toxicological assessment of liver and kidney functions. Therefore, although PSO is traditionally consumed and generally recognized as safe, further rigorous toxicological studies involving hepatic and renal histopathology and functional assays are required to definitively conclude its safety profile in this model. Therefore, the pharmacokinetics, bioavailability, and long‐term safety profile of PSO in chronic disease contexts remain uncharacterized and merit further investigation.

Third, a limitation of the current study is the absence of a standard pharmacological positive control group (e.g., corticosteroids or N‐acetylcysteine). While our findings establish the dose‐dependent efficacy of PSO as a functional food component, further comparative studies are needed to evaluate its potency relative to conventional COPD treatments.

Future studies should aim to address these gaps by incorporating comprehensive molecular assays, extended dosing regimens, and well‐designed clinical trials to evaluate toxicity, efficacy, safety, and mechanistic pathways in human populations. Such efforts will be critical in validating PSO's role as a complementary nutritional strategy in the prevention and management of COPD and other inflammation‐mediated pulmonary disorders.

## Author Contributions

**Sorachai Srisuma:** conceptualization, funding acquisition, methodology, validation, project administration, supervision, resources, writing – review and editing, data curation. **Thanaporn Rungruang:** conceptualization, methodology, validation, visualization, data curation, writing – review and editing, investigation, project administration, supervision. **Gaewarin Liamvilairat:** writing – original draft, formal analysis, investigation, methodology, funding acquisition, data curation, software, project administration, writing – review and editing. **Aikkarach Kettawan:** conceptualization, funding acquisition, writing – review and editing, project administration, visualization, resources, data curation, supervision. **Julalux Thongam:** investigation, software, resources, methodology. **Aurawan Kringkasemsee Kettawan:** investigation, formal analysis, software.

## Funding

This study was supported by the “Chalermphrakiat” Grant (SS, TR) from the Faculty of Medicine Siriraj Hospital, Mahidol University, Thailand.

## Conflicts of Interest

The authors declare no conflicts of interest.

## Supporting information


**Supporting Information: 1** ANOVA table The corresponding *F*‐values, degrees of freedom, and exact *p*‐values for all analyses.

**Supporting Information: 2** *Differential cell counting*: Morphological analysis of cell counts in bronchoalveolar lavage fluid (BALF).

## Data Availability

The data that support the findings of this study are available from the corresponding author upon reasonable request.
