# Supplementary material for: Protective Effect of Thai Perilla frutescens Seed Oil Against Chronic Obstructive Pulmonary Disease Induced by Cigarette Smoke Extract in a Mouse Model
Source: Food Sci Nutr. 2026 Jun 5;14(6):e71994. doi: 10.1002/fsn3.71994 (PMC13241585; doi:10.1002/fsn3.71994)
Supplement: Supplementary file 1 — Supporting Information: 1 ANOVA table The corresponding F‐values, degrees of freedom, and exact p‐values for all analyses. [file FSN3-14-e71994-s001.docx]

**Supplementary report 1 ANOVA table**

| **MLI measurements** | | | |
| --- | --- | --- | --- |
| **ANOVA table** | **DF** | **F (DFn, DFd)** | **P value** |
| Treatment (between columns) | 9 | F (9, 50) = 6.024 | P<0.0001 |
| Residual (within columns) | 50 |  |  |
| Total | 59 |  |  |

**The corresponding F-values, degrees of freedom, and exact *p*-values for all analyses**

| **Total cells count white blood cells in BALF** | | | |
| --- | --- | --- | --- |
| **ANOVA table** | **DF** | **F (DFn, DFd)** | **P value** |
| Treatment (between columns) | 9 | F (9, 35) = 14.99 | P<0.0001 |
| Residual (within columns) | 35 |  |  |
| Total | 44 |  |  |

| **Differential cells count in BALF (Macrophage)** | | | |
| --- | --- | --- | --- |
| **ANOVA table** | **DF** | **F (DFn, DFd)** | **P value** |
| Treatment (between columns) | 9 | F (9, 35) = 13.93 | P<0.0001 |
| Residual (within columns) | 35 |  |  |
| Total | 44 |  |  |

| **Differential cells count in BALF (Eosinophil)** | | | |
| --- | --- | --- | --- |
| **ANOVA table** | **DF** | **F (DFn, DFd)** | **P value** |
| Treatment (between columns) | 9 | F (9, 35) = 4.196 | P=0.0010 |
| Residual (within columns) | 35 |  |  |
| Total | 44 |  |  |

| **Differential cells count in BALF (Neutrophil)** | | | |
| --- | --- | --- | --- |
| **ANOVA table** | **DF** | **F (DFn, DFd)** | **P value** |
| Treatment (between columns) | 9 | F (9, 35) = 1.406 | P=0.2232 |
| Residual (within columns) | 35 |  |  |
| Total | 44 |  |  |

| **Differential cells count in BALF (Basophil)** | | | |
| --- | --- | --- | --- |
| **ANOVA table** | **DF** | **F (DFn, DFd)** | **P value** |
| Treatment (between columns) | 9 | F (9, 35) = 0.5893 | P=0.7968 |
| Residual (within columns) | 35 |  |  |
| Total | 44 |  |  |

| **Total antioxidant activity in serum** | | | |
| --- | --- | --- | --- |
| **ANOVA table** | **DF** | **F (DFn, DFd)** | **P value** |
| Treatment (between columns) | 9 | F (9, 50) = 83.55 | P<0.0001 |
| Residual (within columns) | 50 |  |  |
| Total | 59 |  |  |

| **Total antioxidant activity in BALF** | | | |
| --- | --- | --- | --- |
| **ANOVA table** | **DF** | **F (DFn, DFd)** | **P value** |
| Treatment (between columns) | 9 | F (9, 50) = 57.09 | P<0.0001 |
| Residual (within columns) | 50 |  |  |
| Total | 59 |  |  |

| **The level of MDA in lung tissue** | | | |
| --- | --- | --- | --- |
| **ANOVA table** | **DF** | **F (DFn, DFd)** | **P value** |
| Treatment (between columns) | 9 | F (9, 50) = 68.22 | P<0.0001 |
| Residual (within columns) | 50 |  |  |
| Total | 59 |  |  |
