# Supplementary material for: Protective Effect of Thai Perilla frutescens Seed Oil Against Chronic Obstructive Pulmonary Disease Induced by Cigarette Smoke Extract in a Mouse Model
Source: Food Sci Nutr. 2026 Jun 5;14(6):e71994. doi: 10.1002/fsn3.71994 (PMC13241585; doi:10.1002/fsn3.71994)
Supplement: Supplementary file 2 — Supporting Information: 2 Differential cell counting: Morphological analysis of cell counts in bronchoalveolar lavage fluid (BALF). [file FSN3-14-e71994-s002.docx]

**Supplementary report 2**

***Differential cell counting*:**

**Morphological analysis of cell counts in bronchoalveolar lavage fluid (BALF)**

**50 μm**

| **50 μm** |  | **50 μm**  **50 μm** | 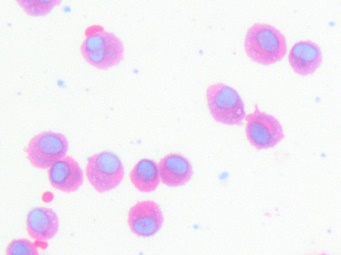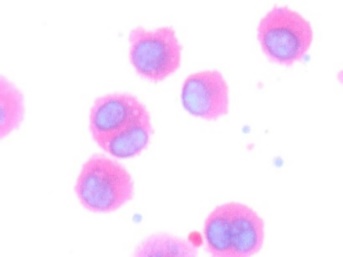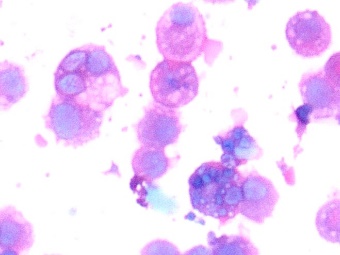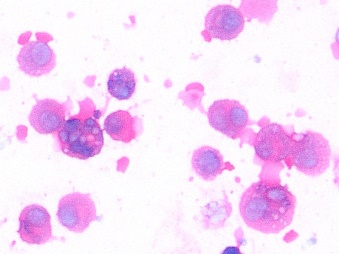 |
| --- | --- | --- | --- |
| Macrophage | Eosinophil | Neutrophil | Basophil |

* Representative images of cell count; macrophage (black arrow), eosinophil (red arrow), neutrophil (green arrow), and basophil (blue arrow).

The differential cell counts were performed using standard cytological staining criteria commonly applied in murine BALF analysis. This picture represents morphological characteristics used for identification of eosinophils, neutrophils, and macrophages.

- Macrophage: Large cells with abundant cytoplasm and a round to kidney-shaped (reniform) nucleus; cytoplasm may appear vacuolated or foamy.
- Neutrophil: Medium-sized granulocyte characterized by a multilobed (segmented) nucleus and pale cytoplasm containing fine, inconspicuous granules.
- Eosinophil: Granulocyte with a banded, horseshoe-shaped, or ring-shaped nucleus and prominent eosinophilic (pink) cytoplasmic granules. (Note: In mice, eosinophils commonly exhibit a ring-shaped nucleus, which differs from the typical bilobed nucleus seen in humans.)
- Basophil: Granulocyte containing coarse, densely basophilic (dark purple-blue) cytoplasmic granules that often partially obscure a lobulated or S-shaped nucleus.
